# Scheduling Independent Partitions in Integrated Modular Avionics Systems

**DOI:** 10.1371/journal.pone.0168064

**Published:** 2016-12-12

**Authors:** Jinchao Chen, Chenglie Du, Pengcheng Han

**Affiliations:** Department of Computer Science, Northwestern Polytechnical University, Xi’an, China, 710072; West Virginia University, UNITED STATES

## Abstract

Recently the integrated modular avionics (IMA) architecture has been widely adopted by the avionics industry due to its strong partition mechanism. Although the IMA architecture can achieve effective cost reduction and reliability enhancement in the development of avionics systems, it results in a complex allocation and scheduling problem. All partitions in an IMA system should be integrated together according to a proper schedule such that their deadlines will be met even under the worst case situations. In order to help provide a proper scheduling table for all partitions in IMA systems, we study the schedulability of independent partitions on a multiprocessor platform in this paper. We firstly present an exact formulation to calculate the maximum scaling factor and determine whether all partitions are schedulable on a limited number of processors. Then with a Game Theory analogy, we design an approximation algorithm to solve the scheduling problem of partitions, by allowing each partition to optimize its own schedule according to the allocations of the others. Finally, simulation experiments are conducted to show the efficiency and reliability of the approach proposed in terms of time consumption and acceptance ratio.

## Introduction

With the growing complexity of modern aircrafts, avionics systems have to integrate and manipulate numerous sensors, actuators and controllers while maintaining high quality of safety and reliability [1]. The traditional federated architecture, which has limited resource sharing and huge power consumption, becomes inappropriate for the design of large-scale avionics systems [2]. A new approach, known as Integrated Modular Avionics (IMA) architecture, has been widely adopted by the avionics industry.

IMA architecture supports the independent development of the various real-time avionics applications on a shared computing platform [3], and enables all applications to be executed within partitions that are spatially and temporally segregated [4]. The spatial segregation implies that each partition has its own system resources, which cannot be accessed by the tasks in other partitions. The temporal segregation specifies that each partition uses a pre-allocated time window to execute all tasks in it. With this partition mechanism, IMA architecture guarantees that the tasks running in different partitions would not overlap with each other [5].

In an IMA system, each partition is a program unit of applications and can be characterized by a computation time and a period. Partitions are executed periodically and exactly, which means the time duration between any two successive instances of a partition is the same and equal to its period. Since a processor may host several partitions running with different periods, the system designers need to design a proper schedule, according to which all partitions subject to the non-overlapping constraints: no two partitions’ instances can overlap during any time period [6]. In other words, the designers have to provide an accurate start time and processor allocation for each partition, such that there is no overlapping time unit among the executions of partitions.

Continuous advancements in avionics have been made and the avionics systems become more and more complex. Faced with the significantly increasing number of partitions, the system designers gradually tend to rely on decision-making tools to produce valid scheduling tables for IMA systems. Meanwhile, for the avionics company, it is preferable to reallocate the start times and processors for all partitions only, rather than to redesign and rebuild the entire system [7]. Hence, it is desirable to develop an effective method, which determines whether all partitions in an IMA system are schedulable and provides valid allocations if they are schedulable.

The multiprocessor scheduling problem of partitions in an IMA system is very challenging. Not only the non-preemption property of partitions’ execution gives it a large-scale computational complexity [8], but also the strict periodicity constraint compounds the difficulty in obtaining the boundary scheduling conditions. In this paper, in order to simplify the problem, we assume that all partitions are independent. The communication links, in the form of execution chains between partitions [1], is not discussed. The main objective of our research is to address two aspects appearing in the process of system design:

1. How to determine whether all independent partitions in an IMA system are schedulable?
2. If an IMA system composed of independent partitions is schedulable, how to allocate valid start time and processor to each partition?

Since the partitions can be modeled as non-preemptive tasks with strict periods, the real-time scheduling problem of independent partitions is classified as non-preemptive and strictly periodic scheduling problems [1], which have been proved to be NP-Hard in the strong sense [9] and only have polynomial time approximation algorithms [10].

Real-time scheduling problem is a fundamental issue in providing guarantees for temporal feasibility of task execution, and widely studied in large-scale systems such as Internet of Things [11, 12] and Cyber-Physical Systems [13, 14]. Significant efforts have been made to provide efficient methods to solve the scheduling problem. Based on fuzzy theory and a genetic algorithm, Shojafar et al. [15, 16] presented a hybrid job scheduling approach to assign jobs with reducing total execution time and execution cost in cloud computing. Using the gravitational emulation local search algorithm, Hosseinabadi et al. [17] proposed a novel algorithm to solve the job-shop scheduling problem in Small and Medium Enterprises. However, in all of those works, the periods of tasks were not strict and some slack time was allowed between successive instances of a periodic task. To the best of our knowledge, there are three types of solutions focusing on the scheduling problem of strictly periodic tasks.

The first type of solutions evolves from the schedulability analysis of strictly periodic tasks and has some special constraints that sharply restrict the range of applications. Korst et al. [8] solved the scheduling problem on two tasks with strict periods, and provided a necessary and sufficient schedulability condition, which had been proved to be a sufficient condition [18] for more than two tasks. Eisenbrand et al. [7, 19] considered the problem on a minimum processor platform and presented an asymptotic approximation schemes with a constraint that all periods were harmonic, i.e., for any two tasks, the period of one task is a multiple of that of the other one. Later, Marouf and Sorel [20] gave a scheduling heuristic based on the constraint that the period of new task had a multiple relationship with those of the existing tasks.

The second type of solutions is based on the critical scaling factor, i.e., the largest possible change for all task computation times [21]. Al Sheikh et al. [1] calculated the critical scaling factor by a best-response algorithm, and used the value calculated to determine whether all partitions were schedulable on a limited number of processors. Pira and Artigues [22] did a similar work and gave a new heuristic to solve the problem with a propagation mechanism for non-overlapping constraints.

The third type of solutions is using the maximum permissible computation time that a new task can have when it is schedulable. Chen et al. [23] represented a task by its eigentask (i.e., setting its worst case computation time to 1), and proved that the maximum permissible computation time of a new task was the largest length of consecutive scheduling slots for its eigentask. If the worst case computation time of the new task was not large than the value calculated, it was determined schedulable. However, this solution does not take into account the dynamic change of the offset and processor allocations of all tasks and has a low scheduling success ratio.

Through schedulability analysis, this paper presents a new approach to solve the schedulability problem of independent partitions in IMA systems. The contributions of our work are as follows.

First, we model the independent partitions as non-preemptive and strictly periodic tasks, and present an exact formulation based on Mixed Integer Linear Programming (MILP) [24] to represent the schedulability constraints of an IMA system and calculate the maximum scaling factor for partitions. If the maximum scaling factor calculated is not less than 1, the partitions are schedulable.

Second, with a Game Theory analogy, we design an efficient heuristic to solve the multiprocessor scheduling problem, by allowing each partition to optimize its own strategy according to the current strategies of the others. This heuristic not only determines the schedulability of an IMA system, but also provides a valid start time and processor allocation for each partition.

The proposed approach has a wide range of applications and can be adapted to partitions with both harmonic and non-harmonic periods. It not only guides the development of IMA systems, but also improves the robustness of a design subject to future changes. We compare our approach with the existing solutions, and show its efficiency and reliability from several aspects.

An earlier version [25] of this paper was presented at the 2015 IEEE International Conference on Progress in Informatics and Computing (PIC 2015). This paper improves the previous conference publication in two aspects:

1. This paper states the multiprocessor scheduling problem of independent partitions, and proposes an exact resolution based on MILP formulation to calculate the maximum scaling factor and determine the schedulability of partitions.
2. This paper analyzes the limitations of our approach, and conduct simulation experiments to compare the performances of our approach, MILP formulation and EMTA algorithm [23] in terms of time consumption and acceptance ratio.

The rest of the paper is organized as follows. Section 2 gives the notations and the strictly periodic partition model used in this paper. Section 3 analyzes the schedulability problem of independent partitions and proposes its MILP formulation. Section 4 presents a heuristic inspired from Game Theory to calculate the maximum scaling factor and determine whether all partitions are schedulable. Section 5 shows the simulation experiments and results. Finally, Section 6 presents the conclusions of this paper and the directions for future work.

## Notations and System Model

**Fig 1:**
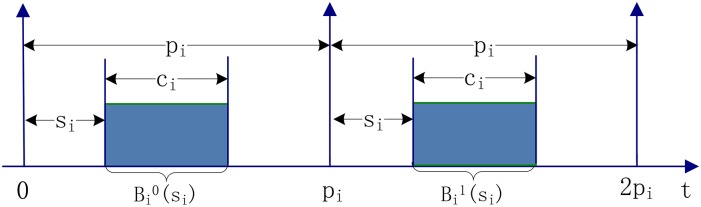
Partition model with strict period.

In this paper, we consider an IMA system composed of *m* identical processors on which a set of *n* partitions *T* = {*τ*_1_, *τ*_2_, …, *τ*_*n*_} requires to be non-preemptively scheduled. Each partition is independent and with strict periodicity constraint. We use a quadruple *τ*_*i*_ = 〈*c*_*i*_, *p*_*i*_, *s*_*i*_, *a*_*i*_〉 to characterize the partition *τ*_*i*_. *c*_*i*_ and *p*_*i*_ are the computation time and the period of *τ*_*i*_. *s*_*i*_ is its offset (i.e., start time of the first instance), and *a*_*i*_ is its assignment (i.e., the processor to which the partition is assigned). When the assignment of *τ*_*i*_ is unknown, a triple *τ*_*i*_ = 〈*c*_*i*_, *p*_*i*_, *s*_*i*_〉 is used to denote the partition *τ*_*i*_. We assume that the partitions’ attributes (i.e., *p*_*i*_, *c*_*i*_, *s*_*i*_ and *a*_*i*_) are all integers. Fig 1 describes an example of a strictly periodic partition used in this paper.

A partition *τ*_*i*_ generates one instance with the computation time *c*_*i*_ at every time unit *s*_*i*_ + *kp*_*i*_ for all *k* ≥ 0. Each instance needs to be executed immediately after its generation and finished before the beginning of *s*_*i*_ + *kp*_*i*_ + *c*_*i*_ without any other partitions’ interrupt, which can be characterized by an open time interval [*s*_*i*_ + *kp*_*i*_, *s*_*i*_ + *kp*_*i*_ + *c*_*i*_). Let $B_{i}^{k}\left(s_{i}\right)$ characterize the time interval occupied by the *k*th (*k* ≥ 0) instance of *τ*_*i*_. According to the strict periodicity constraint, the *k*th instance of *τ*_*i*_ will start at *s*_*i*_ + *kp*_*i*_ and end before the beginning of *s*_*i*_ + *kp*_*i*_ + *c*_*i*_. Therefore, in the strict periodic partition model, there is: $B_{i}^{k}\left(s_{i}\right)=\left[s_{i}+kp_{i},s_{i}+kp_{i}+c_{i}\right)$. Example 1 shows the time units used by two partitions.

**Fig 2:**
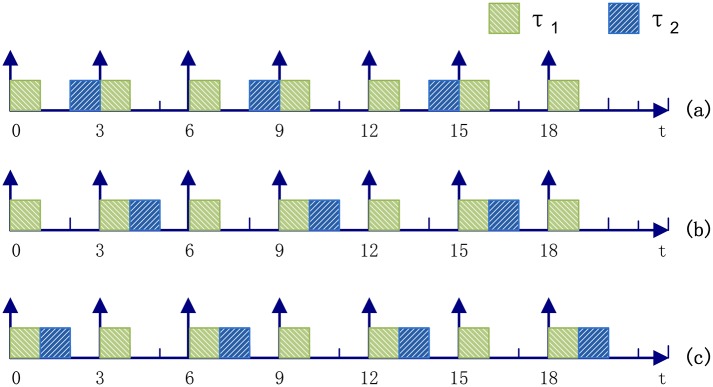
Non-overlapping execution of two periodic partitions on the same processor.

**Example 1.** *Consider a partition set* *T* = {*τ*_1_, *τ*_2_}, *τ*_1_ = 〈1, 3, 0〉 *and* *τ*_2_ = 〈1, 6, *s*_2_〉. *From* Fig 2(a) and 2(b), *we know* *s*_2_ = 2 *or* *s*_2_ = 4 *ensures that partitions* *τ*_1_ *and* *τ*_2_ *would not overlap in a time interval* [0, 17], *which is their recycle time units. To be more exact*, ∀*s*_2_ ∈ {1, 2, 4, 5}, *τ*_1_ *and* *τ*_2_ *can be executed on the same processor without overlapping*. Fig 2(c) *shows the two partitions’ execution when* *s*_2_ = 1, *and now* $B_{2}^{k}\left(s_{2}\right)=\left[6\times k+1,6\times k+2\right)$.

We use *m* to denote the number of identical processors in the IMA system. In this paper, partitions can be allocated to any processor as long as their instances do not overlap in time. We use *T*_*p*_ to represent the partitions allocated to the processor *p* (1 ≤ *p* ≤ *m*), and use $T_{p}^{-i}$ to denote all partitions in *T*_*p*_ except *τ*_*i*_, i.e., $T_{p}^{-i}=T_{p}\setminus \left\{\tau_{i}\right\}$. Meanwhile, we use *g*_*i*,*j*_ to represent the greatest common divisor of the periods of any two partitions *τ*_*i*_ and *τ*_*i*_, i.e., *g*_*i*,*j*_ = *GCD*(*p*_*i*_, *p*_*j*_). Table 1 summarizes the basic notations used in this paper.

**Table 1:** Notations used in this paper.

Symbol	Description
*T*	the partition set to be scheduled
*n*	the number of partitions in *T*
*τ*_*i*_	the *i*th partition in *T*
*c*_*i*_	the computation time of *τ*_*i*_
*p*_*i*_	the period of *τ*_*i*_
*s*_*i*_	the offset of *τ*_*i*_
*a*_*i*_	the assignment of *τ*_*i*_
$B_{i}^{k}\left(s_{i}\right)$	the time interval used by the *k*th instance of *τ*_*i*_
*m*	the number of identical processors in the system
*T*_*p*_	the partitions allocated to the processor *p*
$T_{p}^{-i}$	the partitions allocated to the processor *p* except *τ*_*i*_
*g*_*i*,*j*_	the greatest common divisor of the periods of *τ*_*i*_ and *τ*_*j*_

## Schedulability Problem and Its MILP Formulation

In this section, we analyze the schedulability of partitions in a multiprocessor IMA system. We firstly introduce a schedulability condition for two partitions allocated to the same processor. Then we state the schedulability problem of independent partitions and investigate an exact MILP formulation to provide a determination of whether all partitions are schedulable on a limited number of processors.

### 3.1 Schedulability Analysis for Two Partitions

As we pointed out in Section 2, the *k*th instance of *τ*_*i*_ is executed in the time interval

$$\begin{array}{c} B_{i}^{k}\left(s_{i}\right)=\left[s_{i}+p_{i},s_{i}+kp_{i}+c_{i}\right) \end{array}$$ (1)

If two partitions *τ*_*i*_ and *τ*_*j*_ are schedulable on the same processor, there is no overlapping time unit among their instances. This can be expressed as:

$$\begin{array}{c} \forall k,l\geq 0,B_{i}^{k}\left(s_{i}\right)\cap B_{j}^{l}\left(s_{j}\right)=\emptyset . \end{array}$$ (2)

Although Condition (2) is a necessary and sufficient condition, it could not be applied directly in solving the schedulability problem of two partitions. This is because Condition (2) requires calculating the time intervals occupied by all instances. However, the instances of a partition will be regenerated in every cycle and their number is infinite. The following theorem which gives a more efficient condition, was first proposed by Korst et al. [8] and also had been proven in Al Sheikh et al. [26] and in Chen et al. [23].

**Theorem 1.** *Two partitions* *τ*_*i*_ = 〈*c*_*i*_, *p*_*i*_, *s*_*i*_〉 *and* *τ*_*j*_ = 〈*c*_*j*_, *p*_*j*_, *s*_*j*_〉 *are schedulable on the same processor if and only if*

$$\begin{array}{c} c_{i}\leq \left(s_{j}-s_{i}\right)mod\left(g_{i,j}\right)\leq g_{i,j}-c_{j} \end{array}$$ (3)

We can observe that Condition (3) works for two partitions at a time and cannot be used for multiple partitions. It is difficult to directly give a determination of whether all partitions are schedulable. We solve this problem by adopting the concept of *scaling factor* [21], which represents the possible change for the computation times of all partitions.

The scaling factor *λ* is an easily recognized sign of the schedulable state of an IMA system. For example, Al Sheikh et al. [1] calculated the scaling factor based on game theoretic approach, and used it to determine whether all partitions in a set were schedulable. If *λ* ≥ 1, the partition set was considered to be schedulable upon a limited number of processors; otherwise, more processors were required.

### 3.2 Exact Formulation

In this section, we propose an MILP formulation for calculating the maximum scaling factor on a multiprocessor platform, and use the scaling factor calculated to determine whether the system is schedulable.

**Fig 3:**
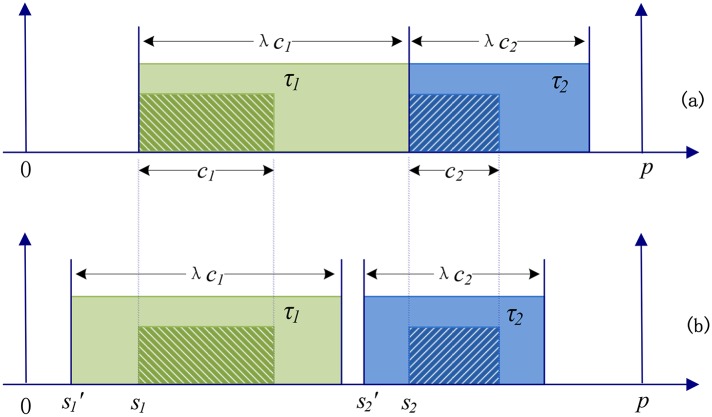
Impact of the scaling factor *λ* on the computation times of two partitions.

We firstly analyze the extension process of partition computation times when they are scaled. Fig 3 illustrates the impact of *λ* on the computation times of two partitions assigned to the same processor. Hashed rectangles represent the initial time units occupied by the first instances of the two partitions, whereas the larger filled ones represent the scaled time budgets. Fig 3(a) extends the computation times of two partitions according to the method proposed by Al Sheikh et al. [1], in which the start times of the instances remain the same but the end times change in accordance with the scaling factor. However, as shown in Fig 3(b), the extension process discussed in this paper is different. The computation time of each instance is equally extended from the center to both the left and right side. This means that the centers of the computation time units remain the same; but the start times and end times of the instances are changed when the scaling factor *λ* is not equal to 1.

Since all computation times are scaled by *λ* proportionally, for any partition *τ*_*i*_ (1 ≤ *i* ≤ *n*), the value of its computation time is changed to *λc*_*i*_. We use $s_{i}^{\prime }$ to denote the start time of *τ*_*i*_ after its computation time has been scaled. From Fig 3(b) we know, $2\left(s_{i}-s_{i}^{\prime }\right)+c_{i}=\lambda c_{i}$, which yields $s_{i}^{\prime }=s_{i}-\left(\lambda -1\right)c_{i}/2$. Condition (3), which is used to determine whether two original partitions are schedulable on the same processor, should be updated to

$$\begin{array}{c} \lambda c_{i}\leq \left(s_{j}^{\prime }-s_{i}^{\prime }\right)mod\left(g_{i,j}\right)\leq g_{i,j}-\lambda c_{j} \end{array}$$ (4)

Now we analyze the non-overlapping constraints on a multiprocessor platform. When all partitions in the set *T* are schedulable on *m* processors, there are two constraints: (1) Each partition should be allocated to one and only one processor; (2) The instances of any two partitions allocated to the same processor cannot overlap during any time period.

We use a *n*-row *m*-column vector $\vec{a}=\left(a_{i,k}\right)$ (1 ≤ *i* ≤ *n* and 1 ≤ *k* ≤ *m*) to represent the allocations of all partitions. Each variable *a*_*i*,*k*_ has a Boolean value and denotes whether the partition *i* is allocated to the processor *k*. If the partition *i* is allocated to the processor *k*, *a*_*i*,*k*_ = 1; otherwise, *a*_*i*,*j*_ = 0, i.e.,

$$\begin{array}{c} a_{i,k}=\{\begin{array}{cc} 1 & \text{if}\;\tau_{i}\;\text{is assigned to the processor}\;k\\ 0 & \text{otherwise} \end{array} \end{array}$$

The first non-overlapping constraint requires allocating each partition to one and only one processor. That is to say, the sum of every row of the vector $\vec{a}$ is equal to 1, i.e.,

$$\begin{array}{c} \forall i\in \left[1,n\right],\sum_{1\leq k\leq m}a_{i,k}=1 \end{array}$$

When two partitions *τ*_*i*_ and *τ*_*j*_ are allocated to the same processor *k*, their offsets should satisfy Condition (4). Hence, the second non-overlapping constraint can be expressed as:

$$\begin{array}{ccc} & & \forall i,j\in \left[1,n\right],\forall k\in \left[1,m\right],a_{i,k}=a_{j,k}=1,i\neq j\\ & & \lambda c_{i}\leq \left(s_{j}^{\prime }-s_{i}^{\prime }\right)mod\left(g_{i,j}\right)\leq g_{i,j}-\lambda c_{j} \end{array}$$ (5)

The modulo operation (*mod*) in Condition (5) is not linear. In order to use it in linear programming, the modulo operation should be transformed to

$$\begin{array}{c} \left(s_{j}^{\prime }-s_{i}^{\prime }\right)mod\left(g_{i,j}\right)=\left(s_{j}^{\prime }-s_{i}^{\prime }\right)-g_{i,j}\times e_{i,j} \end{array}$$ (6)

where $e_{i,j}=\left\lfloor \frac{s_{j}^{\prime }-s_{i}^{\prime }}{g_{i,j}}\right\rfloor$. The factor *e*_*i*,*j*_ is a new integer variable representing the quotient from the modulo operation *mod*. Since $0\leq s_{i}^{\prime }\leq p_{i}-\lambda c_{i}$ and $0\leq s_{j}^{\prime }\leq p_{j}-\lambda c_{j}$, the value of *e*_*i*,*j*_ ranges from $\frac{\lambda c_{i}-p_{i}}{g_{i,j}}$ to $\frac{p_{j}-\lambda c_{j}}{g_{i,j}}$. Therefore, Condition (5) becomes

$$\begin{array}{ccc} & & \forall i,j\in \left[1,n\right],\forall k\in \left[1,m\right],a_{i,k}=a_{j,k}=1,i\neq j\\ & & \lambda c_{i}\leq \left(s_{j}^{\prime }-s_{i}^{\prime }\right)-g_{i,j}\times e_{i,j}\leq g_{i,j}-\lambda c_{j}\\ & & \frac{\lambda c_{i}-p_{i}}{g_{i,j}}\leq e_{i,j}\leq \frac{p_{j}-\lambda c_{j}}{g_{i,j}} \end{array}$$

The calculation of the maximum scaling factor is seeking optimal offset and assignment allocations for all partitions, such that the largest possible change in the partition computation times can be affordable to satisfy the non-overlapping constraints. The exact formulation can be written as the following program:

$$\begin{array}{ccc} \; & \text{maximum}\;\lambda & \\ & \text{subject to}\\ & \;\;\forall i\in \left[1,n\right],\forall k\in \left[1,m\right],a_{i,k}\in \left\{0,1\right\} \end{array}$$ (7)

$$\begin{array}{cc} & \;\;\forall i\in \left[1,n\right],s_{i}^{\prime }\in \left[0,p_{i}-\lambda c_{i}\right] \end{array}$$ (8)

$$\begin{array}{cc} & \;\;\forall i\in \left[1,n\right],\sum_{1\leq k\leq m}a_{i,k}=1 \end{array}$$ (9)

$$\begin{array}{cc} & \;\;\forall i,j\in \left[1,n\right],\forall k\in \left[1,m\right],a_{i,k}=a_{j,k}=1,i\neq j\\ & \;\;\lambda c_{i}\leq \left(s_{j}^{\prime }-s_{i}^{\prime }\right)-g_{i,j}\times e_{i,j}\leq g_{i,j}-\lambda c_{j}\\ & \;\;\frac{\lambda c_{i}-p_{i}}{g_{i,j}}\leq e_{i,j}\leq \frac{p_{j}-\lambda c_{j}}{g_{i,j}} \end{array}$$ (10)

Constraints Eqs (7) and (8) show the range restrictions of the offsets and assignments of the partitions. Condition (9) represents the allocation constraint that concerns assigning each partition to one and only one processor. Condition (10) is the non-overlapping constraint of each two partitions assigned to the same processor.

The exact MILP formulation discussed above seeks the maximum scaling factor by searching all possible offset and assignment allocations for the partitions, which is exceedingly laborious and time-consuming. Inspired from Game Theory [27], a highly efficient heuristic is proposed in the following section.

## Best Response Algorithm

In this section, we propose an approximation algorithm to calculate the maximum scaling factor, determine the schedulability of all partitions, and provide valid allocations if the partitions are schedulable. This algorithm is inspired from Game Theory, which is the study of strategic decision making. In a game, each player should make the best action according to the current known strategies of the other players. All players take turns in fixed order to adapt their strategies until no better action can be made. Think of partitions as players and their strategies are the changes of their offsets and assignments. All partitions take turns to select their offsets and assignments such that their computation times can be scaled as much as possible. This kind of solution is called the best response solution, which was firstly introduced by Al Sheikh et al. [1, 26], and also studied by Pira and Artigues [6, 22].

We firstly optimize the center (i.e., the middle time point of the first instance) of one partition *τ*_*i*_ on a given processor *p* such that *τ*_*i*_ has the largest common scaling factor with all partitions allocated to the processor *p*. Then on a multiprocessor platform, we try to find the best assignment besides the best center for *τ*_*i*_ to ensure that *τ*_*i*_ has the largest scaling factor *λ*_*i*_, by which the computation times of *τ*_*i*_ and all partitions assigned to the same processor with *τ*_*i*_ can be multiplied. Finally, partitions take turns to optimize their centers and assignments according to mostly known allocations until an equilibrium is reached. When this calculation stops, the maximum scaling factor is the minimum value of the factors of all partitions, i.e., *λ* = min_1≤*i*≤*n*_*λ*_*i*_. With this scaling factor calculated, this algorithm gives a determination of whether all partitions are schedulable and provides valid offset and assignment allocations for partitions if they are schedulable.

### 4.1 Best Center Procedure on a Given Processor

For each partition *τ*_*i*_ (1 ≤ *i* ≤ *n*), we design a best center procedure *BC*(*i*, *p*) to find an optimal center *o*_*i*_ such that the computation times of *τ*_*i*_ and all other partitions can be scaled by the largest factor. The offsets, assignments and centers of other partitions remain the same.

Since *o*_*i*_ is the center of *τ*_*i*_, $o_{i}=s_{i}+\frac{c_{i}}{2}=s_{i}^{\prime }+\frac{\lambda c_{i}}{2}$. Meanwhile, *e*_*i*,*j*_ is the quotient from the modulo operation in Condition (4), i.e., $e_{i,j}=\left\lfloor \frac{s_{j}^{\prime }-s_{i}^{\prime }}{g_{i,j}}\right\rfloor$. Putting the variables *o*_*i*_ and *e*_*i*,*j*_ into Condition (4), we get

$$\begin{array}{ccc} & & \lambda c_{i}\leq \left(s_{j}^{\prime }-s_{i}^{\prime }\right)mod\left(g_{i,j}\right)\leq g_{i,j}-\lambda c_{j}\\ & & \Rightarrow \lambda c_{i}\leq \left(s_{j}^{\prime }-s_{i}^{\prime }\right)-g_{i,j}\times e_{i,j}\leq g_{i,j}-\lambda c_{j}\\ & & \Rightarrow \lambda c_{i}\leq \left(o_{j}-\frac{\lambda c_{j}}{2}-o_{i}+\frac{\lambda c_{i}}{2}\right)-g_{i,j}e_{i,j}\leq g_{i,j}-\lambda c_{j}\\ & & \Rightarrow \lambda c_{i}-\frac{\lambda c_{i}}{2}+\frac{\lambda c_{j}}{2}\leq o_{j}-o_{i}-g_{i,j}e_{i,j}\leq g_{i,j}-\lambda c_{j}-\frac{\lambda c_{i}}{2}+\frac{\lambda c_{j}}{2}\\ & & \Rightarrow \frac{\lambda (c_{i}+c_{j})}{2}\leq o_{j}-o_{i}-g_{i,j}e_{i,j}\leq g_{i,j}-\frac{\lambda (c_{i}+c_{j})}{2}\\ & & \Rightarrow \frac{\lambda (c_{i}+c_{j})}{2}\leq \left(o_{j}-o_{i}\right)mod\left(g_{i,j}\right)\leq g_{i,j}-\frac{\lambda (c_{i}+c_{j})}{2}\\ & & \Rightarrow \lambda \leq \min(\frac{2\left(o_{i}-o_{j}\right)mod\left(g_{i,j}\right)}{c_{i}+c_{j}},\frac{2(g_{i,j}-\left(o_{i}-o_{j}\right)mod\left(g_{i,j}\right))}{c_{i}+c_{j}}) \end{array}$$

We use $\lambda_{i,j}^{p}$ to denote the largest scaling factor for *τ*_*i*_ and any partition *τ*_*j*_ assigned to the processor *p*. Therefore,

$$\begin{array}{c} \lambda_{i,j}^{p}=\min(\frac{2\left(o_{i}-o_{j}\right)mod\left(g_{i,j}\right)}{c_{i}+c_{j}},\frac{2(g_{i,j}-\left(o_{i}-o_{j}\right)mod\left(g_{i,j}\right))}{c_{i}+c_{j}}) \end{array}$$

$\lambda_{i,j}^{p}$ represents the factor by which the computation times of *τ*_*i*_ and any other *τ*_*j*_ can be scaled without violating the schedulability constraint. Now we extend *τ*_*j*_ to all the partitions assigned to the processor *p* except *τ*_*i*_ (i.e., $\tau_{j}\in T_{p}^{-i}$), and use $\lambda_{i}^{p}$ to denote the largest scaling factor that the computation times of all partitions can be multiplied by. Hence,

$$\begin{array}{c} \lambda_{i}^{p}=\min_{\tau_{j}\in T_{p}^{-i}}\lambda_{i,j}^{p} \end{array}$$ (11)

For each valid value of *o*_*i*_, there is a corresponding factor $\lambda_{i}^{p}$ for the partitions assigned to the processor *p* while keeping *τ*_*i*_ schedulable with all other partitions. We use $m\lambda_{i}^{p}$ to denote the largest permissible scaling factor when only *τ*_*i*_ can change its center freely on the processor *p*. There is:

$$\begin{array}{c} m\lambda_{i}^{p}=\max_{0\leq o_{i}\leq p_{i}}\lambda_{i}^{p}=\max_{0\leq o_{i}\leq p_{i}}\min_{\tau_{j}\in T_{p}^{-i}}\lambda_{i,j}^{p} \end{array}$$ (12)

Now we analyze the upper bound on the largest permissible scaling factor $m\lambda_{i}^{p}$ such that the best center procedure can stop when the calculated value reaches this upper bound. We can observe that the value of $\lambda_{i,j}^{p}$ only changes with *o*_*i*_. Only when $\left(o_{i}-o_{j}\right)mod\left(g_{i,j}\right)=\frac{g_{i,j}}{2}$, $\lambda_{i,j}^{p}$ reaches its maximum value, which means $\lambda_{i,j}^{p}\leq \frac{g_{i,j}}{c_{i}+c_{j}}$. Putting this condition into Eq (12), we get an upper bound on $m\lambda_{i}^{p}$:

$$\begin{array}{c} m\lambda_{i}^{p}\leq \min_{\tau_{j}\in T_{p}^{-i}}\frac{g_{i,j}}{c_{i}+c_{j}} \end{array}$$ (13)

The best center procedure *BC*(*i*, *p*) performs this calculation and stops after all valid values of *o*_*i*_ have been considered or the scaling factor calculated reaches the upper bound. Its pseudo-code is shown in Algorithm 1.

**Algorithm 1:** Best center procedure *BC*(*i*, *p*)

  **Input:** *τ*_*i*_ and a processor *p*

  **Output:** the permissible scaling factor $m\lambda_{i}^{p}$, and the best center *bo*_*i*_ for *τ*_*i*_

1  $m\lambda_{i}^{p}\leftarrow -1$; *bo*_*i*_ ← −1;

2  $T_{p}^{-i}\leftarrow T_{p}\setminus \left\{\tau_{i}\right\}$;

3  $u_{i}^{p}\leftarrow \min_{\tau_{j}\in T_{p}^{-i}}\frac{g_{i,j}}{c_{i}+c_{j}}$;

4  **for** *k* = 0 to *p*_*i*_ **do**

5   *o*_*i*_ ← *k*; *t* ← *p*_*i*_/*c*_*i*_;

6   **foreach** $\tau_{j}\in T_{p}^{-i}$ **do**

7    $\lambda_{i,j}^{p}=\min(\frac{2\left(o_{i}-o_{j}\right)mod\left(g_{i,j}\right)}{c_{i}+c_{j}},\frac{2(g_{i,j}-\left(o_{i}-o_{j}\right)mod\left(g_{i,j}\right))}{c_{i}+c_{j}})$;

8    **if** $\lambda_{i,j}^{p}<t$ **then**

9     $t\leftarrow \lambda_{i,j}^{p}$;

10    **end**

11   **end**

12   **if** $t>m\lambda_{i}^{p}$ **then**

13    $m\lambda_{i}^{p}\leftarrow t$; *bo*_*i*_ ← *k*;

14   **end**

15   **if** $m\lambda_{i}^{p}\geq u_{i}^{p}$ **then**

16    break;

17   **end**

18  **end**

19  **return** ($m\lambda_{i}^{p},bo_{i}$);

Now we analyze the computational complexity of Algorithm 1. The main computation part of Algorithm 1 is from line 4 to 18, which has a structure of double closed loops. The inner loop (from line 6 to 11) at most repeats *n* times, where *n* is the number of partitions in the system. Given the outer loop at most repeats *p*_*i*_ times, the total running time of Algorithm 1 is *O*(*np*_*i*_). If we use *P*_*max*_ to denote the maximum period of all partitions, the running time complexity of Algorithm 1 is *O*(*nP*_*max*_).

### 4.2 Best Response Procedure on a Multiprocessor Platform

In this section, we extend the best center procedure *BC*(*i*, *p*) to a multiprocessor platform, and present a best response procedure *BR*(*r*) to find the best assignment besides the best center for a given partition *τ*_*i*_. The best assignment and the best center guarantee that *τ*_*i*_ has the largest scaling factor according to the current allocations.

From Section 4.1 we know, when *τ*_*i*_ is assigned to the processor *p*, the permissible factor by which the computation times of all partitions can be multiplied, is $m\lambda_{i}^{p}$ and can be calculated by the best center procedure *BC*(*i*, *r*). In order to choose the best assignment, we need to compute the permissible factor on each processor and select the largest one. We use *λ*_*i*_ to denote the maximum permissible scaling factor for *τ*_*i*_ when only the center and assignment of *τ*_*i*_ change on a multiprocessor platform, hence:

$$\begin{array}{c} \lambda_{i}=\max_{1\leq p\leq m}m\lambda_{i}^{p} \end{array}$$ (14)

According to Condition (13), the scaling factor when *τ*_*i*_ optimizes its center on a given processor *p* is not larger than $\min_{\tau_{j}\in T_{p}^{-i}}\frac{g_{i,j}}{c_{i}+c_{j}}$, which means $\lambda_{i}\leq \min_{\tau_{j}\in T,j\neq i}\frac{g_{i,j}}{c_{i}+c_{j}}$. When the calculated value reaches this upper bound, the processors left can be skipped and the best response procedure stops. The pseudo-code for this best response procedure is given in Algorithm 2. Since the best center procedure *BC*(*i*, *p*) has a complexity of *O*(*nP*_*max*_), the complexity of the best response procedure *BR*(*i*) is *O*(*mnP*_*max*_).

**Algorithm 2:** Best response procedure *BR*(*i*)

  **Input:** *τ*_*i*_ in a partition set *T*

  **Output:** the largest factor *λ*_*i*_ for *τ*_*i*_, the corresponding center *bo*_*i*_ and assignment *ba*_*i*_

1  *λ*_*i*_ ← 0; *bo*_*i*_ ← −1; *ba*_*i*_ ← −1;

2  $u\leftarrow \min_{\tau_{j}\in T,j\neq i}\frac{g_{i,j}}{c_{i}+c_{j}}$;

3  **for** *p* = 1 to *m* **do**

4   (*t*, *to*_*i*_)←*BC*(*i*, *p*);

5   **if** *t* > *λ*_*i*_ **then**

6    *λ*_*i*_ ← *t*; *bo*_*i*_ ← *to*_*i*_; *ba*_*i*_ ← *p*;

7   **end**

8   **if** *λ*_*i*_ ≥ *u* **then**

9    break;

10   **end**

11  **end**

12  **return** (*λ*_*i*_, *bo*_*i*_, *ba*_*i*_);

### 4.3 Equilibrium-Based Heuristic

Now we present a heuristic to calculate the maximum scaling factor for the computation times of all partitions based on Game Theory. We think of partitions as players and their strategies are the modification of their centers and assignments. All partitions take turns to use the best response procedure *BR*(*i*) to update their strategies such that their computation times can be scaled as much as possible. When no partition in the set *T* can improve its center or assignment using the best response procedure, an equilibrium state is reached and the iterative process stops. At this time, the maximum scaling factor *λ* is the minimum value of the permissible factors of all partitions, i.e., *λ* = min_1≤*i*≤*n*_*λ*_*i*_. If *λ* ≥ 1, the partitions are schedulable on this multiprocessor platform; otherwise, more processors are required. When *λ* ≥ 1, the values of centers and assignments are valid allocations for all partitions.

We use $\lambda_{i}^{k}$, $o_{i}^{k}$ and $a_{i}^{k}$ to denote the permissible scaling factor, the corresponding center and assignment obtained from the best response procedure *BR*(*i*) when *τ*_*i*_ updates its allocation in the *k*th iteration. As the authors did in Al Sheikh et al. [1] and in Pira and Artigues [22], we assume that *τ*_*i*_ does not change its center or assignment if the best response procedure does not improve its current scaling factor. That is to say: if $\lambda_{i}^{k}\leq \lambda_{i}^{k-1}$, $o_{i}^{k}=o_{i}^{k-1}$ and $a_{i}^{k}=a_{i}^{k-1}$. The pseudo-code for this equilibrium-based heuristic is given in Algorithm 3.

**Algorithm 3:** Equilibrium-based heuristic

  **Input:** Partition set *T* and the number of processor *n*

  **Output:** The maximum scaling factor *λ* for all partitions

1  *k* ← 1;

2  **repeat**

3   **for** *i* = 1 to *n* **do**

4    $\left(\lambda_{i}^{k},bo_{i},ba_{i}\right)\leftarrow BR\left(i\right)$;

5    **if** $\lambda_{i}^{k}>\lambda_{i}^{k-1}$ **then**

6     *o*_*i*_ ← *bo*_*i*_; *a*_*i*_ ← *ba*_*i*_;

7    **end**

8    **else**

9     $o_{i}^{k}\leftarrow o_{i}^{k-1}$; $a_{i}^{k}\leftarrow a_{i}^{k-1}$;

10    **end**

11   **end**

12   *k* ← *k* + 1;

13  **until** *T*^*k*^ = *T*^*k*−1^

14  $\lambda \leftarrow \min_{1\leq i\leq n}\lambda_{i}^{k}$;

15  **if** *λ* ≥ 1 **then**

16   The partitions in *T* are schedulable on this *m*-processor platform;

17  **end**

18  **else**

19   The partitions in *T* are not schedulable on this *m*-processor platform;

20  **end**

According to the Proposition 4 presented in Al Sheikh et al. [26], this heuristic converges and reaches one or more fixed points in at most (n+hh)n iterations where *h* = ⌈*α*_*max*_ Δ^−1^⌉, $\alpha_{max}=\max_{i}\min_{j\neq i}\frac{g_{i,j}}{c_{i}+c_{j}}$ and $\Delta =\min_{j,k}\frac{1}{lcm(c_{j},c_{k})}$. In each iteration, the best response procedure *BR*(*i*) is used to select the best offset and assignment. As we pointed out in Sect. 4.2, the best response procedure *BR*(*i*) runs in *O*(*mnP*_*max*_). Hence, the running time complexity of the heuristic is O(mn2Pmax(n+hh)).

### 4.4 Limitation Analysis

Even though this equilibrium-based heuristic can determine whether all partitions are schedulable upon a limited number of processors and provide a proper offset and processor allocation for each partition, it is not an optimal method and the scaling factor calculated by this heuristic is not the maximum one. This is because our approach is based on Game Theory and stops when equilibrium states are reached. It does not completely search the solution space and some valid allocations would be skipped by our approach. Some partition sets that are actually schedulable on the limited number of processors would be rejected and thrown away by our approach. This is one of the reasons why our algorithm has a lower scheduling success ratio than the exact solutions.

## Simulation Results

In this section we conduct simulation experiments to analyze the performance of our approach proposed in Section 4. We compare the experimental results with those of MILP formulation and the assignment algorithm based on eigentask and mapping function (EMTA) proposed in Chen et al. [23]. The machine used has an Intel(R) Core(TM) i5-3320M CPU 2.60GHz and 4.00GB of system memory.

MILP is an exact framework for the linear programs in which some or all variables are required to take integer values. It is solved with the CPLEX Optimizer [28] from IBM ILOG and can completely find a feasible solution for periodic scheduling problem if no time limit is set. EMTA is a first fit algorithm to allocate the partitions one by one and obtain the numbers of processors required by the partition sets.

The generation procedure of partition sets is the same as that described in Chen et al. [23]. First the UUnifast-Discard algorithm [29] was adopted to generate the utilization *u*_*i*_ (1 ≤ *i* ≤ *N*) for each task. Then, a random value *p*_0_ was chosen from 5 to 9 as a base integer, i.e., *p*_0_ = *U*[5, 9]. Next, for non-harmonic partitions, periods were chosen uniformly from the set {2^*x*^3^*y*^5^*z*^ *p*_0_: *x*, *y*, *z* ∈ [0, 4]}, as was derived from Eisenbrand et al. [19]. For harmonic partition, a period ratio *k*_*i*_ (1 ≤ *i* ≤ *N*) was selected randomly from [1, 6], and periods were constructed as *p*_*i*_ = *k*_*i*_ *p*_*i*−1_. Finally, the computation time of each task was given by *c*_*i*_ = ⌈*p*_*i*_ *u*_*i*_⌉. We analyze the performance of our approach in terms of time consumption and acceptance ratio.

### 5.1 Time Consumption Evaluation

**Fig 4:**
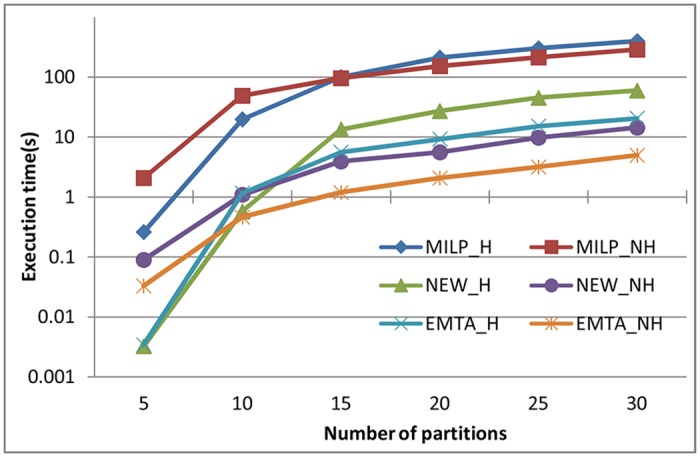
Execution times required by MILP formulation, our approach and EMTA algorithm to determine the schedulability of the partitions.

With a logarithmic scale, Fig 4 shows the execution times required to determine whether the partition sets are schedulable. The partition sets were generated when the system utilization was 1.0. The fields of “MILP_H”, “MILP_NH”, “NEW_H”, “NEW_NH”, “EMTA_H” and “EMTA_NH” represent the average execution times required by MILP formulation, our approach and EMTA algorithm when partitions were generated with harmonic and non-harmonic periods. Each point in Fig 4 (also in all figures used in Sect. 5) represents the average value of the experimental results of 100 instances.

We can see that the time consumption of the three solutions has a similar changing tendency that it grows gradually along with the increase of the number of partitions. This is because that the more partitions are tested on, the more possible offset and assignment allocations should be considered to find an optimal solution. When the number of partitions is the same, the time consumption of our approach is a little higher than that of EMTA. This is because EMTA algorithm does not consider the modification of all partitions and has a lower scheduling success ratio. Relatively, our approach requires less time than MILP formulation. When the number of partitions is 10, the execution times required by our approach for harmonic and non-harmonic partitions are 0.6 and 1.1 seconds, which are 30 and 41 times less than those required by the MILP formulation respectively. This demonstrates that our method is faster in analyzing the schedulability of the partitions.

**Fig 5:**
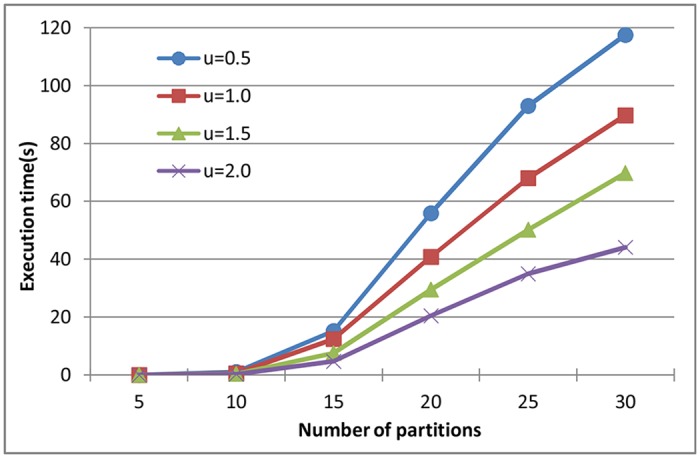
Time consumption of our approach on harmonic partitions generated with different system utilization.

Fig 5 shows the effects of system utilization on the performance of our approach when the partitions are chosen from harmonic periods. We can see that when the number of partitions is fixed, the execution time of our approach decreases along with the increase of the system utilization. The reason is that lower system utilization means shorter partition computation time is constructed, and there are more available time units left for a new partition. More valid offset and assignment allocations for partitions need to be considered to determine the schedulability of the partitions. Hence, lower system utilization means longer time is required when the number of partitions is fixed.

### 5.2 Acceptance Ratio Evaluation

In this section, we compare the performances of MILP formulation, EMTA algorithm and our approach in terms of acceptance ratio, i.e., the percentage of partition sets determined schedulable on a limited number of processors [30]. There exist some partition sets that are rejected by one method, but can be determined schedulable according to other methods. We perform the experiments on the same partition sets, and record the number of partition sets that are successfully scheduled by each method. A more accurate method would have a higher acceptance ratio.

**Fig 6:**
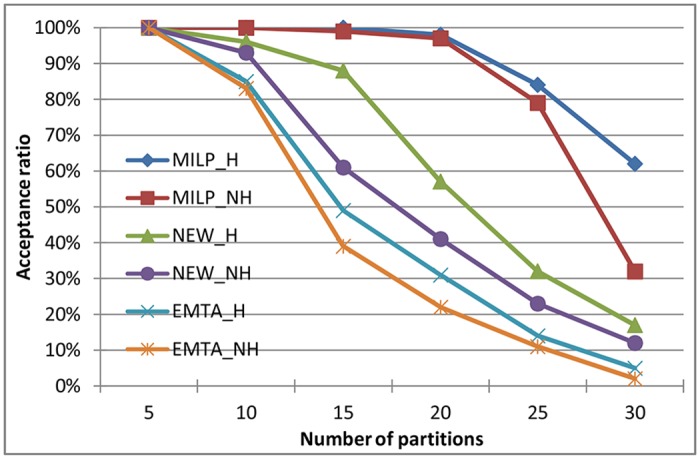
Acceptance ratios of MILP formulation, our approach and EMTA algorithm on partitions with harmonic and non-harmonic periods.

In the experiment of Fig 6, partitions are generated when the system utilization is 1.0 and the number of processors in the system is limited to 4. It demonstrates that the acceptance ratios of the three solutions have a similar changing tendency that they gradually decrease from 100 percent to less than 70 percent along with the increase of the number of partitions from 5 to 30. MILP which is an exact method, has the highest acceptance ratio on a given number of partitions, and our approach has the second highest acceptance ratio. When the number of harmonic partitions is 15, the acceptance ratio of our approach is 8% less than that of MILP but one time more than that of EMTA algorithm.

**Fig 7:**
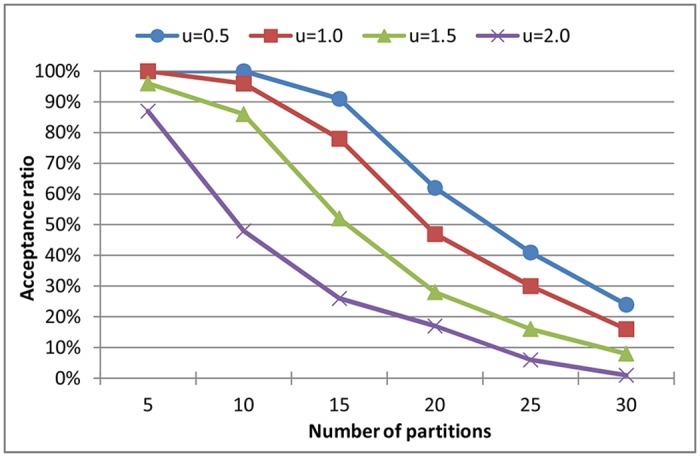
Acceptance ratios of our approach on partitions with harmonic periods.

In the experiment of Fig 7, partitions are chosen from harmonic periods, and we evaluate the acceptance ratios of our approach by tuning the system utilization from 0.5 to 2.0. As can be seen that the acceptance ratios decrease along with the increase of the number of partitions. Meanwhile, when the number of partitions is fixed, the acceptance ratios drop with the system utilization increases. The reason is that with larger system utilization, larger computation times are generated, and less partition sets are determined to be schedulable on a limited number of processors. Hence, larger system utilization leads to a lower acceptance ratio.

From Section 5.1 and 5.2 we can find that the heuristic proposed in this paper is a reasonable compromise between time consumption and acceptance ratio, to solve the scheduling problem of partitions. It is true that the acceptance ratio of the heuristic may be less than that of the exact MILP formulation, but the relative error can be accepted when taking the time cost into account. For example, when the number of harmonic partitions is 15, the acceptance ratio of the heuristic is 88%, and the relative error is 8%. At the same time, the time consumption of MILP is 101 seconds, which is almost 7 times more than that of the heuristic. This heuristic purchases a high speed at the cost of a little of acceptance ratio.

## Conclusions and Further Work

In this paper, we studied the schedulability problem of partitions in IMA systems. The partitions are independent and with strict periods, which means that the time duration between two successive instances of a partition is fixed and no lag is allowed once the partition’s offset and assignment is defined. Through schedulability analysis, this paper proposes an efficient approach to solve the non-preemptive scheduling problem of independent partitions in multiprocessor IMA systems.

We firstly modeled the independent partitions as non-preemptive tasks with strict periods, and used a quadruple to represent the attributes (computation time, period, offset and assignment) of a given partition. Then based on MILP formulation, we proposed an exact approach to incorporates the constraints of an IMA system, and got the maximum scaling factor, by which the computation times of all partitions can be multiplied before the system became unschedulable. Finally, with a Game Theory analogy, we presented an efficient heuristic to calculate the maximum scaling factor and provided a determination of whether the partitions were schedulable. In this heuristic, partitions took turns in selecting the best offsets and assignments according to the most recent known allocations of other ones until an equilibrium state was reached.

The proposed equilibrium-based heuristic has a wide range of applications in IMA systems and can be applicable across partitions with both harmonic and non-harmonic periods. With the maximum scaling factor calculated, this heuristic not only determines whether the partitions are schedulable, but also provides valid allocations for all partitions if they are schedulable upon a multiprocessor real-time platform. It helps the system designers in developing the IMA systems and improves the robustness of a design subject to future changes.

Compared with the exact MILP formulation, this heuristic can achieve feasible solutions with small relative errors in a short amount of time, which means that the heuristic is more efficient in terms of speed. The quality of the heuristic is also shown to handle large scale IMA systems composed of tens of processors and hundreds of partitions, where the MILP formulation cannot find feasible solutions if time limit is set. Meanwhile, since the partitions are modeled as non-preemptive tasks with strict periods, the schedulability analysis proposed in this work can be used to solve the scheduling problem of a strictly periodic task model, which is one of the most frequently models studied in the real-time system area. Hence, the results and heuristic presented in this paper can be used in other application areas and would find applicability in a wide variety of real-time systems.

Although our approach can obtain solutions in a relatively short amount of time, we would like to consider some aggressive notions of approximation to reduce the search space and improve its performance. Meanwhile, we are interested in studying the schedulability of partitions under the constraints of communications, and would like to see whether some results proposed here can be adapted to dependent partitions.
